# A Review on Maternal and Infant Microbiota and Their Implications for the Prevention and Treatment of Allergic Diseases

**DOI:** 10.3390/nu15112483

**Published:** 2023-05-26

**Authors:** Yifan Wu, Gongsheng Zhang, Yucong Wang, Xin Wei, Huanhuan Liu, Lili Zhang, Lanwei Zhang

**Affiliations:** 1 Key Laboratory of Dairy Science, Ministry of Education, College of Food Science, Northeast Agricultural University, Harbin 150030, China; wuyf_hit@hotmail.com (Y.W.);; 2 School of Chemistry and Chemical Engineering, Harbin Institute of Technology, Harbin 150001, China; 3 College of Food Science and Engineering, Ocean University of China, Qingdao 266100, China

**Keywords:** maternal flora, infant flora, probiotics, infant allergic disease

## Abstract

Allergic diseases, which are closely related to the composition and metabolism of maternal and infant flora, are prevalent in infants worldwide. The mother’s breast milk, intestinal, and vaginal flora directly or indirectly influence the development of the infant’s immune system from pregnancy to lactation, and the compositional and functional alterations of maternal flora are associated with allergic diseases in infants. Meanwhile, the infant’s own flora, represented by the intestinal flora, indicates and regulates the occurrence of allergic diseases and is altered with the intervention of allergic diseases. By searching and selecting relevant literature in PubMed from 2010 to 2023, the mechanisms of allergy development in infants and the links between maternal and infant flora and infant allergic diseases are reviewed, including the effects of flora composition and its consequences on infant metabolism. The critical role of maternal and infant flora in allergic diseases has provided a window for probiotics as a microbial therapy. Therefore, the uses and mechanisms by which probiotics, such as lactic acid bacteria, can help to improve the homeostasis of both the mother and the infant, and thereby treat allergies, are also described.

## 1. Introduction

Allergic diseases, including food allergy, atopic dermatitis (AD), asthma, and allergic rhinitis, pose a serious threat to a baby’s growth and health, as well as worry parents [1]. When an infant’s immune system is not well developed, some antigens cross the infant’s fragile epithelial barrier and trigger inappropriate responses from the immune system [2]. The ratio of T helper (Th) 1/Th 2 cells as well as the ratio of regulatory T (Treg)/Th17 cells was reduced in the allergic infants [3]. Consequently, the under-expressed Treg cells and over-expressed Th2 cells generate excess immunoglobulin E (IgE) and inflammation, which are the main inducing factors for infant allergic diseases [4].

Numerous studies show that allergic diseases and related immune disorders in infants are closely related to the infant and maternal flora. Studies on the differences in flora between allergic and healthy infants have focused on the infant gut flora, as mounting evidence over the past decades has described the gut flora characteristics of allergic infants [5]. Meanwhile, the maternal flora not only influences the infant development during the perinatal period but can also be transferred through breast milk, which is the only recommended food source for infants after birth, thereby contributing to the establishment of the infant’s early gut flora and the development of the infant’s immune system [6]. Increasing evidence suggests that maternal flora is associated with infant allergic diseases and can predict the onset of allergies in childhood.

Here, a brief description of the mechanisms of infant allergies was undertaken with highly cited classic articles by searching the PubMed database. We further focused our review on the characteristics of maternal and infant flora associated with allergic diseases since the advent of next-generation sequencing (NGS) in 2006 by searching the PubMed database using the host-flora-, infant-, and allergic-diseases-related key words between 2010 and 2023. By compiling representative cohort studies with standardized enrolment settings and broad coverage, the compositional and metabolic differences in maternal and infant flora associated with allergic diseases are discussed. Subsequently, the therapeutic implications and related mechanisms of lactic acid bacteria (LAB) in allergic diseases were also highlighted by summarizing the extensively researched anti-allergic LAB through clinical and animal trials in PubMed from 2010 to 2023. We expect to link the changes in the host flora associated with allergic diseases to microbial therapy and to provide impetus for the treatment of allergic diseases in infants.

## 2. Allergic Diseases and Differentiation of T Lymphocytes

**Figure 1:**
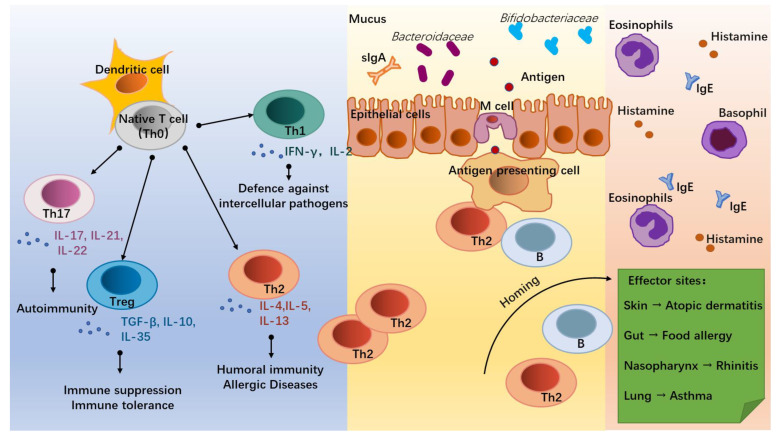
Infant allergic diseases and T helper (Th) cell differentiation. Under the influence of the external environment and host conditions, native T cells (Th0) activated by dendritic cells (DC) differentiate into different subsets to regulate immune homeostasis. Of these, Th1 cells secrete IFN-γ and IL-2, which mediate defense against intercellular pathogens. Th2 cells (secrete IL-4, IL-5, and IL-13) mediate humoral immunity and allergic responses. Th17 cells (secrete IL-17, IL-21, and IL-22) mediate autoimmunity. Treg cells (secrete TGF-β, IL-10, and IL-35) mediate immune suppression and the development of immune tolerance. In allergic infants, accompanied by dysbiosis of gut flora and hyperpolarization of Th2 cells, some antigens cross the intestinal epithelium and interact with antigen-presenting cells, Th2 cells, and B cells. Through homing, Th2 and B cells trigger inflammatory responses at effector sites by promoting the recruitment of eosinophils and basophils, the secretion of Immunoglobulin E (IgE), and the release of histamine, thereby triggering allergic diseases such as atopic dermatitis, food allergy, rhinitis, and asthma.

The onset of allergic diseases is associated with the differentiation of T lymphocytes (T cells, Figure 1). Under the co-stimulation with the surrounding environment, dendritic cells (DC) induce the native T cells (Th0) into distinct T helper subsets mainly including Th1, Th2, Th17, and Treg cells [7]. Of these, Th2 cells, which secrete Interleukin (IL)-4, IL-5, and IL-13, are the main mediators of humoral immunity and the key cells in triggering an allergy [8]. Due to insufficient stimulation by external pathogens during infancy, the balance tilts towards Th2 cells, leading to the development of an allergy. With increasing age and exposure to external pathogens, some infants are able to self-heal their allergic symptoms and achieve a normalized immune function [9,10,11]. Th1 cells are mainly involved in cellular immunity and participate in the defense against intercellular pathogens by producing INF-γ and IL-2 [12], working in antagonism with Th2 cells [13]. Th17 cells can act on neutrophils and induce autoimmunity in vivo [14] and are observed to be increased in the body of allergic patients with disturbed immune homeostasis through the secretion of IL-17, IL-21, and IL-22 [15,16]. While Treg cells are regulated by the intestinal environment and microbiota, they are expressed in the lymphoid tissues of the intestine to down-regulate the expression of Th2 cells and establish food-protein-related immune tolerance by producing anti-inflammatory cytokines, such as IL-10 and TGF-β [17]. 

The effects of microbial stimuli may help to adjust the decreased ratio of Th1/Th2 cells in infants with allergic disorders. Microorganisms can induce the establishment of adaptive immunity through bacterial endotoxins, lipopolysaccharide (LPS) receptors, and Toll-like receptors on antigen-presenting cells (APC) and induce Th1 differentiation through the release of cytokines, such as IL-12 and IL-18, via the NF-kB pathway [18,19]. Similarly, mycobacterial antigens can induce Th1 cell differentiation while inhibiting the Th2 cell expression [20]. For the Treg cells that trigger immune tolerance, some intestinal helminths such as *Strinoidogyne* can induce Treg secretion in mice, and thus protect against colitis in a model of colitis [21], while disruption of the intestinal flora and antibiotic drugs reduce Treg cell differentiation in the intestine of mice [21]. Metabolites of commensal bacteria such as *Bacteroides fragilis* polysaccharides are also involved in the induction of Treg cell differentiation and promote the development of immune tolerance [22]. For Th17 cells, which are involved in immunity to exogenous pathogens, such as *K. pneumoniae*, *S. aureus*, *S. enteritidis serum*, and *S. flexneri* [14,23], their differentiation can be induced by segmented filamentous bacterium (SFB) [24].

Allergic diseases in infants are associated with inappropriate immune responses to allergens. The Th0 cells are abnormally directed toward the differentiations of Th2 and Th17 cells, promoting the humoral immunity and inflammation of the infants and leading to the occurrence of allergic diseases [4,23]. Before the infant’s immune system is fully matured, allergen proteins are processed by Microfold (M) cells in the terminal ileum through DC presentation, allowing Th2 cells to receive an initial signal generated by the interaction of the TCR-CD3 complex and a co-stimulatory signal between CD28 on T cells and the B7 family on APC, causing an allergic sensitization response [25]. Activated T and B lymphocytes then migrate by “homing” to specific target organs, such as the gastrointestinal tract, respiratory system, skin, and central nervous system, and release inflammatory factors, such as histamine, eosinophils, and basophils, to cause different allergic symptoms, such as diarrhea, asthma, and eczema [26].

## 3. Maternal Microbiota and Infant Allergy

### 3.1. Breast Milk Flora

**Figure 2:**
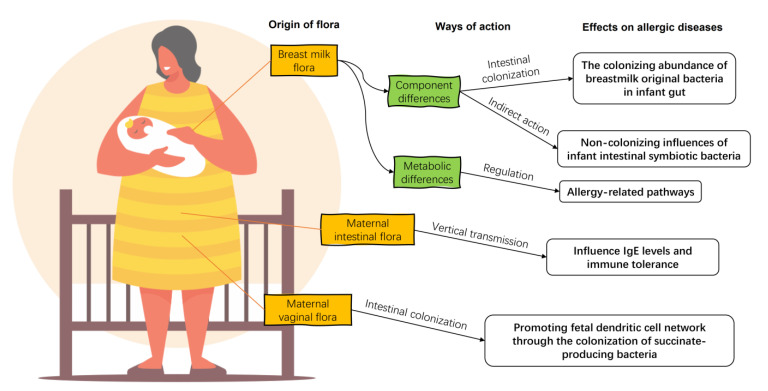
The impacts of maternal flora on infant allergic diseases. Maternal breast milk flora enters directly into the gut of the allergic infant, regulating the composition of the infant intestinal flora and the diversity of associated strains directly and indirectly. There are differences in the ability of the breast milk flora of allergic infants to metabolize their hosts compared to healthy ones. The maternal gut flora influence offspring IgE levels and immune tolerance by vertical transmissions. Maternal vaginal bacteria regulate the development of the offspring’s dendritic cell network by colonization.

Breast milk is the preferred food for infants recommended by modern medicine, and its composition has a significant impact on the health of infants and young children. For a long time, human milk was considered to be aseptic. However, after the advent of sequencing technology, pyrosequencing of the DNA encoded by the V1–V2 hypervariable region of 16S rRNA of human milk flora confirmed that human milk contains microorganisms other than skin-contaminated microorganisms [27]. Breast milk microorganisms, along with oligosaccharides, immunoglobulins, and other organic matter, temporarily replace the infant’s gut flora and help the infant establish its initial gut microbiota and immune system (Figure 2) [28], including promoting the production of antibody Secretory Immunoglobulin A (sIgA), regulating lymphocyte function, promoting mucosal growth, and regulating cytokines [29].

The sources of breast milk flora include maternal intestinal flora, the oral cavity flora, and the infant’s oral cavity flora, with a bacterial content of 10^6^ [6]. Specific bacterial species in the maternal body can be transferred to breast milk through the mammary gland. In the analysis of breast tissue from some women with mastectomies, bacteria such as *Actinobacter*, *Stenotrophomonas*, *Pseudomonas*, *Streptococcus*, and *Staphylococcus* were found in the otherwise sterile breast, which were also the dominant bacteria in the breast milk flora observed in subsequent studies [30]. The increased intestinal permeability of the woman during pregnancy provides a pathway for the delivery of the gut bacteria into the breast milk. At this time, the maternal intestinal bacteria can be transferred to the breast milk through the lamina propria under the joint action of DCs and CD18+ cells [31]. Postpartum, when the infant is sucking on the breast milk, the suction force creates a large amount of counter-suction in the breast, promoting the exchange of microbiota, such as *Staphylococci* and *Streptococci*, between the infant’s oral cavity and the mammary gland [32]. Bacterial transport pathways within the mother’s body and feedback from the infant’s oral cavity during breastfeeding provide opportunities for infants to influence the composition of the breast milk flora. Therefore, the composition of the breast milk flora and the relative abundance of each component change with the growth of the infant [33]. The composition of the breast milk flora is influenced not only by the mother’s diet [34] and body weight [33], but also by the infant’s mode of delivery [33,35] and gender [30]. 

The breast milk flora consists mainly of *Streptococcus* and *Staphylococcus*, with a high individual variation and a much higher flora diversity than the infant gut flora. The composition of the breast milk flora has significant regional differences, which reflects the geographical differences in the incidence of allergic diseases [36]. The dominant species in the breast milk flora in the Pullman region of the United States include *Streptococcus*, *Staphylococcus*, *Serratia*, and *Corynebacteria* [27], which is similar to the breast milk flora of Canadian mothers with similar lifestyles and geographic environments. The dominant bacteria in the breast milk flora of Canadian mothers includes *Staphylococcus*, *Pseudomonas*, *Edwardsiella*, *Pantoea*, *Treponema*, *Streptococcus*, and *Campylobacter* [37]. In turn, sequencing of the V1-V3 region of 16S rRNA in breast milk from the Netherlands in Europe revealed that in addition to *Streptococcus* and *Staphylococcus*, *Leuconostocaceae* and *Lactobacillaceae* were also abundant in the breast milk flora of Dutch mothers [38]. Common symbiotic bacteria in the human gut, such as *Propioibacterium* and *Escherichia*, were also observed in breast milk flora, but their percentage was less than 1%. In addition, symbiotic bacteria, such as *Campylobacter*, *Corynebacterium*, and *Candidatus*, whose effects on the host are not fully understood, are also present in the breast milk flora [30].

There is not as much research on the relationship between breast milk flora and allergic diseases in infants, but the existing research has shown the importance of breast milk flora. Compared to healthy breast milk, the composition of breast milk flora in infants with allergic diseases differs in its transmission to offspring and its regulation of host metabolism. On the one hand, breast milk flora may influence the development of the infant gut flora directly through colonization, as the relative abundance of *Lachnospira*, *Veillonella*, *Faecalibacterium*, and *Rothia*, which are homologues of breast milk, is significantly reduced in the intestine of infants with asthma [39]. On the other hand, the sparCC network analysis, which reveals the correlations among taxa [40], showed that some bacteria in breast milk flora which cannot be colonized in the intestine or can only exist in small amounts are able to regulate the infant gut microbiota by influencing gut colonizing bacteria, such as the influence of *Streptophyta* on *Enterobacteriales* and *Gemellales* on *Actinomycetales* and *Lactobacilliales* [41].

In the analysis of the characteristics of breast milk flora fed to eczema infants less than 6 months old, the relative abundance of *Bacillales*, *Weissella*, *Leuconostocaceae*, and *Flavobacterium* in the flora of breast milk fed to eczema infants was higher than that in healthy breast milk flora, and the relative abundance of *Verrucomimicrobiaceae*, *Xanthomonadaceae*, *Sphingomonadaceae*, and *Moracellaceae* was higher in healthy breast milk flora [41]. For food allergies, the relative abundance of *Acinetobacter* and *Pseudomonas* are higher in the breast milk flora of the food allergy group, while the relative abundance of *Escherichia*, *Prevotella*, *Bifidobacterium*, *Clostridium*, *Veillonella*, *Roseburia*, *Akkermansia*, *Ruminococcus*, *Lachnospiraceae*, *Clostridium* XlVa, and *Blautia* are higher in the breast milk flora of the healthy group [42]. Differences in flora composition will lead to differences in microbe–host modulation. The indicator bacterium *Bifidobacterium bifidum* in healthy breast milk has higher glycolytic activity towards glycans, which is a property that positively regulates immune system homeostasis [43]. In addition, *Akkermansia*, a member of the *Murecleiaceae* family, which is widely present in the breast milk of healthy infants, is considered an emerging and promising probiotic with a positive regulatory effect on obesity and inflammatory response [44]. For allergy-related breast milk flora, the characteristic bacterium *Bacillus* in eczema breast milk is reported, as some of them have potential pathogenicity [45]. 

The overall metabolic capacity of the breast milk flora of allergic infants on the host is also different from that of the healthy breast milk flora. The pathways of antigen processing and presentation, Th17 cell differentiation, IL-17 signaling, and steroid hormone biosynthesis were less well regulated by eczematous breast milk flora than by healthy breast milk flora based on a simulation analysis according to colony composition [41]. All of these differential pathways are associated with allergic and inflammatory responses. Steroids are common anti-inflammatory agents [46]. Antigen presentation by commensal flora contributes to the development of immune tolerance [47]. The function of Th17 is not fully understood, but Th17 and the associated IL-17 may promote intestinal barrier integrity via the Mincle–Syk axis [48]. Thus, the flora in breast milk for allergic diseases exhibits weaker immune stimulation and associated regulation of metabolism and may be closely related to the development of allergic disease in infants.

### 3.2. Maternal Vaginal and Gut Flora

Although not as continuously involved in the development of the infant’s intestinal system as the breast milk flora, the mother’s vaginal flora contributes to the development of immune tolerance in the infant through vertical transmission. No significant differences in the vaginal flora of mothers of allergic infants were found. However, in a comparative analysis of the infant fecal flora and the corresponding vaginal flora of the mothers of allergic infants, it was found that *Bifidobacterium*, *Lactobacillus*, and *G. vaginalis* in the vaginal flora can be transferred to the infant gut and persist early in life, as well as influence detectable levels of IgE in infants in the subsequent development. Of these, *L. jensenii* may contribute to the development of immune tolerance during allergic inflammation in a vertically transmitted manner [49].

Maternal gut microbiota can promote the development of the infant’s intestinal immune system during pregnancy by providing metabolites and substrates necessary for fetal growth [50]. The composition of the maternal gut flora may also predict the risk of allergic disease in infants and young children. Increases in maternal aerobes and enterococci are associated with the incidence of asthma in infants and young children, but not with eczema or atopic rhinitis [51]. The beneficial bacteria carried in the maternal gut, such as *Prevotella copri*, can significantly reduce the risk of food allergy in offspring [52]. *Prevotella* can stimulate the function and migration of DC cells through the production of succinate, promote the establishment of the fetal DC network, and confer protective effects against allergic diseases and asthma [53]. Although an exchange of skin flora may occur between mothers and their infants during daily breastfeeding and parenting, there are no reports on the effect of maternal skin flora other than the breast on allergic diseases in infants.

## 4. Infant Flora and Infant Allergic Diseases

### 4.1. Infant Gut Flora

The gut is the largest immune organ in the body and is inhabited by numerous microorganisms that exchange metabolites and signals in the gut, forming a microbial ecological network that regulates the homeostasis of the body [54].

The intestinal flora begins to colonize within 2 h of birth and transforms into adult types by the age of three, which is one of the most important factors in the immune development of an infant. The lack of gut flora in germ-free mice results in a defect in the development of lymphoid tissue in the spleen, thymus, and lymph nodes. The reduced expression of CD4^+^ cells in the lamina propria, reduced secretion of sIgA from the intestinal surface, and reduced Peyer’s patches were also observed in the germ-free mice [55]. Furthermore, these incomplete immune developments show both a skewing of CD4+ Th0 cell differentiation towards Th2 cells and an over-accumulation of constitutive natural killer cells (iNKT), leading to the susceptibility to allergic diseases and colitis. However, when introduced into the germ-free mouse gut, commensal gut bacteria promote B cell development in the Peyer’s patches, increase sIgA production and secretion, and strengthen the intestinal mucosal immune barrier [56]. Symbiotic bacteria can also regulate Th1/Th2 cell differentiation by inducing Th1 cell differentiation, achieving an immune balance between the two [57]. Animal studies have also shown that the effects of early gut flora colonization can persist into adulthood. Lack of early gut flora stimulation in germ-free mice leads to elevated serum IgE levels, increasing the risk of food allergies in adulthood, whereas the colonization of young mice with symbiotic gut flora before 4 weeks of age improves the immune imbalance in germ-free mice in adulthood and returns serum IgE to normal levels [58].

**Figure 3:**
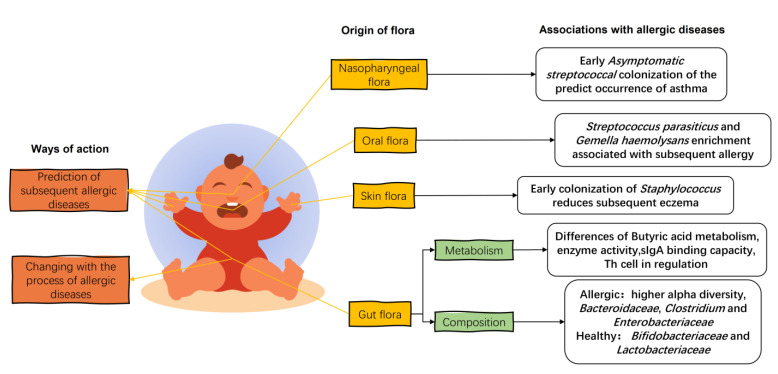
The effects of infant flora on infant allergic diseases. The infant gut flora changes with the stage of allergy, and the early colonization of specific strains of the infant’s gut, oral, skin, and nasopharyngeal flora influences the later development of allergy in childhood. Organic acid metabolism, enzymatic activity, and immune regulation in the gut flora of allergic infants are characteristic. The intestinal flora of allergic infants showed a higher relative abundance of the *Bacteroidaceae*, *Clostridium*, and *Enterobacteriaceae* and a lower relative abundance of the *Bifidobacteriaceae* and *Lactobacteriaceae*.

In the first few weeks of life, the gut flora consists of *Enterobacteriaceae*, *B. fragilis*, *Staphylococcus*, *Enterococcus*, and *Bifidobacterium*, with a predominance of parthenogenetic anaerobes and a gradual evolution towards specialized anaerobes [59]. When allergic diseases occur in infants, the gut flora of allergic infants is disturbed and differs significantly from the diversity and composition of the healthy infant gut flora (Figure 3). The fecal flora of infants with eczema up to 6 months of age has a higher alpha diversity than that of healthy infants [41]. This was also seen in the gut flora of mice transplanted with the feces of cow’s-milk-allergic infants [60]. The establishment of an anaerobic environment in the gut and the ingestion of food after birth in infants under one year of age can exert positive pressure on the development of the gut flora, leading to a reduction in alpha diversity and the establishment of a healthy gut flora [61]. This higher alpha diversity in the infants with allergic diseases may be due to the incomplete development of the intestinal tract, resulting in a weaker selection of commensal flora [62] and leading to a decrease in the colonization of beneficial bacteria and an increase in the colonization of weedy bacteria [63]. 

In terms of flora composition, the relative abundance of *Bacteroidaceae*, *Clostridium*, and *Enterobacteriaceae* in the gut of allergic children is higher compared to healthy infants, while the relative abundance of *Bifidobacteriaceae* and *Lactobacteriaceae* is lower [5]. An analysis of fecal collections from allergic and non-allergic 2-year-old young children in Estonia and Sweden showed that allergic children had reduced abundance of *Bifidobacteriaceae* and *Lactobacteriaceae* and an increased abundance of *E. coli* and *S. aureus* in the gut, with a high proportion of *Bacteroides* and low proportion of *Enterobacteriaceae* [5,64]. Long-term changes in the flora of allergic children have been summarized in a follow-up study of infants with allergies. A short-term reduction in the relative abundance of *Ruminococcus* and a long-term reduction in the relative abundance of *Bacteroides*, *Prevotella*, and *Coprococcus* were observed. In addition, the metabolism and colonization capacity of Bifidobacteria in the gut of infants with allergic diseases is also impaired compared to that of healthy infants, suggesting that not only the composition of the flora but also the metabolism of species is different in the gut flora of allergic infants [62].

The allergic flora disorders are also prospective and indicative. A prospective trial showed that alterations in the flora structure of allergy-prone children at around 1 year of age already preceded the onset of an allergy, with these infants having a low *Bifidobacterium*/*Clostridium* ratio in the infant gut at 3 weeks of age. An increased abundance of *Staphylococcus aureus* and a sustained decrease in *Bifidobacterium* were also observed at 6 months of age [65]. Changes in gut flora in early infancy are also associated with the ability of an allergy to self-heal in adulthood. In a study following 226 children with cow’s milk allergy, those who were able to spontaneously resolved the allergy at the age of 8 years had increased abundance of both *Clostridium* and *Firmicutes* between 3 and 6 months of age and had reduced fatty acid metabolism during this process, compared with those children who did not resolve the allergy in adulthood [64]. 

A healthy gut flora protects the host from allergic diseases, whereas the gut flora of allergic hosts induces allergic reactions. Butyric acid-producing *Clostridium* spp. fecal anaerobic rod-shaped bacteria in the gut flora of healthy infants protect the host from allergic symptoms in mice [66]. After transplantation of fecal flora from patients with different grades of atopic dermatitis (AD) into the intestines of mice and sensitizing the mice to AD, it was found that mice inoculated with gut flora from patients with high grades had more pronounced dermatitis symptoms and higher serum expression levels of the pro-inflammatory factors IL-1β, TNF-α, IL-4, IL-5, and IL-6 [67]. The same situation was observed in the fecal flora of cow’s-milk-allergic infants, where feces from cow’s-milk-allergic and healthy infants were transplanted into germ-free mice and orally sensitized with whey protein; the mice transplanted with the gut flora of cow’s-milk-allergic infants showed diarrheal symptoms and higher levels of Th2-type immune response and Th17 response [56,68]. As allergic symptoms subside, the gut flora of allergic infants changes accordingly. A study of the differences in gut microbiota between allergic and non-allergic infants aged 2–12 months in Spain at the time of allergy diagnosis and after 6 months of treatment with hydrolyzed formula powder found that allergic infants had higher total bacterial counts and anaerobic bacteria but lower levels of yeast. After six months of treatment with hydrolyzed formula powder the composition of the flora was changed, as the number of anaerobic bacteria and lactobacilli was increased, while the percentage of bifidobacteria and enterobacteria was decreased [60]. 

Differences in gut flora composition are accompanied by the regulation of host metabolism, as evidenced by the induction of Treg cell differentiation, the production and catabolism of butyric acid in the gut, and the ability to bind sIgA. Symbiotic bacteria in early infants induce Tgfb1 gene expression in Tregs to drive the differentiation of the long-surviving RORγt^+^ Treg, thereby regulating tolerance to bacterial and dietary antigens in the gut, whereas dysbiosis of the gut flora impairs RORγt+ Treg cell differentiation and the establishment of the intestinal barrier [69]. The butyric acid producer *A. caccae* of the genus *Clostridium* in the gut flora of healthy infants protects the host against allergic symptoms in mice [66]. 

The enzyme activity of the gut flora of allergic infants is also lower than that of healthy infants. The gene abundance of carbohydrate-activating enzymes (CAZyme) that degrade butyric acid and human milk oligosaccharides (HMO) is reduced in the gut flora of 3-month-old allergic infants, while the gene abundance of carbohydrate-active enzymes that degrade resistant starch in the microbiota of allergic infants at 1 year old is also reduced [70]. In terms of interactions with host immune factors, the proportion of intestinal microbiota that binds to breast-milk-derived sIgA is lower in 12-month-old children with asthma than in healthy infants [71].

### 4.2. Oral, Skin, and Nasopharyngeal Flora of Infants

In addition to the intestinal flora, the infant’s oral flora is closely related to the breast milk flora, and its maturity is an indicator of early and subsequent allergy development. A 7-year study showed that oral microbial diversity in allergic infants was consistently lower than in healthy infants. The relative abundance of *Prevotella* and *Neisseria mucosa/sicca/flava* increased at 3 months in healthy children compared to children who developed an allergy during the first 7 years, whereas *S. parasanguinis* and *G. haemolysans* were more abundant in allergic children at the same age. At 6 months, the abundance of *Bacteroidales* and *C. matruchotii* increased in children who remained healthy, whereas *Streptococcus salivarius*/*cristatus*/*vestibularis* and *Selenomonas* sp. were more abundant at the onset of allergy [72].

The composition and diversity of the skin flora changed over time and was not significantly affected by the method of birth or feeding, nor was it associated with the development of eczema. However, early colonization of the skin flora with *Staphylococcus* reduced the incidence of subsequent eczema. Twelve-month-old infants with AD before the onset of eczema were not colonized by *S. aureus*, and early skin colonization by commensal *Staphylococcus* at 2 months reduces the risk of AD in infants at 1 year of age [73].

The infant nasopharyngeal microbiome is associated with the severity of lower respiratory tract infections and with the risk of developing asthma. A culture of *S. pneumoniae*, *H. influenzae*, *M. catarrhalis*, and *S. aureus* in hypopharyngeal aspirates from 1-month-old infants revealed that children neonatally colonized with *S. pneumoniae*, *H. influenzae*, and *M. catarrhalis* had elevated blood eosinophil counts and total IgE in serum at 4 years of age and an increased risk of recurrent wheezing and asthma early in life [74]. Early asymptomatic *Streptococcal* colonization of the nasopharynx is a strong predictor of asthma, and *Streptococcal* colonization of the nasopharyngeal microbiome during the asymptomatic period in infants with chronic asthma at less than 5 months of age is associated with subsequent chronic asthma, with subsequent asthma risk levels negatively correlated with age at the time of initial *Streptococcal* colonization. Targeting pathogenic bacteria in the nasopharyngeal microbiome is considered a preventive approach for asthma [75].

## 5. LAB Intervention on Host Flora and Treatment of Allergic Diseases

### 5.1. Mechanism of LAB Intervention on Host Flora

**Figure 4:**
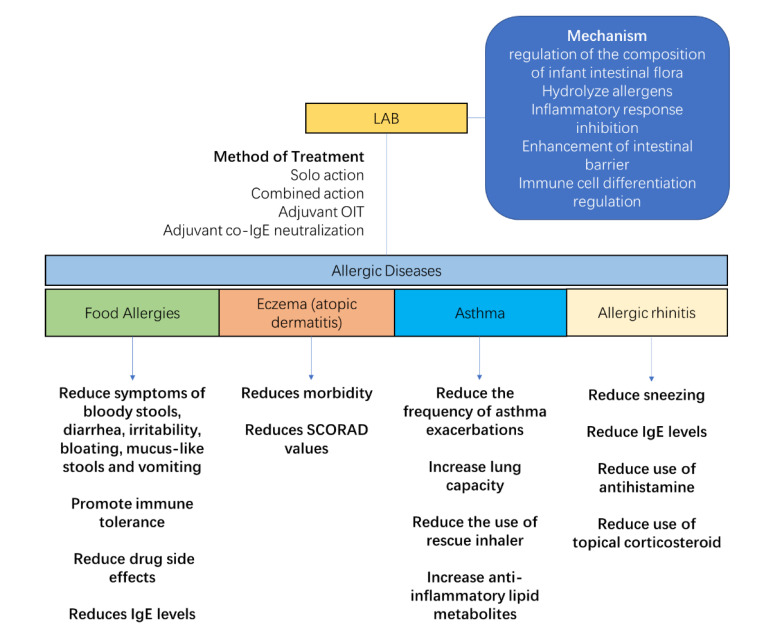
Lactic acid bacteria (LAB) relieve the symptoms of allergic diseases. Their anti-allergic effect is achieved through five areas: regulation of the intestinal flora of allergic infants, hydrolysis of allergens, inhibition of the inflammatory response, enhancement of the intestinal barrier, and modulation of immune cell differentiation. Therapeutic modalities include solo action, combined action, and adjuvant therapy. For allergic diseases, LAB reduce digestive disorders caused by food allergy, increase immune tolerance, reduce the side effects of drug administration, reduce the morbidity and symptoms of eczema, increase lung function and immune tolerance in asthmatics, and reduce symptoms and the use of medication of rhinitis.

The regulation of maternal and infant flora helps to prevent and alleviate allergic diseases (Figure 4). LAB, as a common symbiotic bacterium with immunomodulatory properties, has been shown to be effective in regulating infant allergies in numerous clinical studies. The main functions of LAB in allergy relief are to regulate the composition of the gut flora, hydrolyze allergens, inhibit the allergy-induced inflammatory response, improve the intestinal barrier, and regulate immune cell differentiation. The use of a syngeneic preparation composed of *B. infantis* and HMO, which successfully colonizes the adult gut without the use of antibiotics, promotes butyrate production and improves allergic relief against the corresponding dysbiosis [76]. For the combination of *L. rhamnosus* GG (LGG) with hydrolyzed protein formula powder, the relative levels of *Blautia*, *Roseburia*, *Coprococcus*, and butyrate-producing bacteria in food allergic infants were increased in the gut of cow’s milk allergy (CMA)-allergic infants. In addition, good colonization ability is an important factor in the effectiveness of probiotics. The colonization of LGG was 10 times more effective than the control bacteria *L. rhamnosus* GR-1, and therefore better colonized the nasal cavity of mice to act and prevent allergies [77].

LAB possess a good ability to degrade protein allergens. During the fermentation of yogurt, the antigenicity of β-lactoglobulin in cow’s milk was reduced by using *Lactobacillus* [78]. *L. acidophilus*, *S. thermophilus*, and *L. delbrueckii* ssp. *Bulgaricus* can sustainably hydrolyze the bovine milk protein allergen β-lactoglobulin [79]. Meanwhile, supplementation with bifidobacteria is able to provide the intestine with digestive enzymes for the degradation of large protein antigens, such as phosphoprotein phosphatase, a degradation enzyme of α-casein [80]. On the other hand, in the regulation of the intestinal environment, the binding of LAB to intestinal epithelial cells blocks the pathway of inflammatory factor production in target cells initiated by the entry of I-kB into the nucleus to suppress inflammation [54]. *Bifidobacterium* and *Lactobacillus* bind specifically to intestinal mucosal epithelial cells to form a bacterial membrane structure that strengthens the intestinal mucosal barrier, reduces the permeability of the intestinal wall, increases the stability of the immune system, and resists the harmful effects of external allergenic substances on the organism [81]. At the same time, the intestinal colonization of these LAB can also inhibit the apoptosis of epithelial cells to increase the production of mucin and sIgA, prevent the adhesion of pathogens, and enhance the barrier effect of the intestinal mucosa [82]. 

In addition, LAB can modulate the number of DC cells, NK cells, and Treg cells by acting on the host’s intestinal immune system, thereby reducing inflammation and allergic diseases. There are a variety of ways in which LAB can regulate the differentiation of immune cells. Some LAB can inhibit the formation of the allergic environment by entering Peyer’s lymph patches on the intestinal epithelial surface, regulating the proliferation and activation of DC cells, and promoting the production of TNF-α and IL-6 by mature DCs and the differentiation of native T cells to Th1 cells [83,84]. *B. bifidum* has a regulatory effect on Treg cell differentiation, as oral supplementation of mice with *B. shortum* before weaning promotes the proliferation of FoxP3+ Treg cells and inhibits the inflammatory response [85]. *L. fermentum* CECT5716 and *L. salivarius* CECT5713 induce the expansion of NK cells in innate immunity and also have an activating effect on the moderate differentiation of CD4+ and CD8+ T cells and Treg cells in adaptive immunity [86]. Among them, *L. salivarius* CECT5713 is able to suppress lipopolysaccharide-induced inflammatory responses by stimulating IL-10 production by splenocytes, while *L. fermentum* CECT5716 can counteract inflammatory responses by altering the Th1/Th2 ratio through the induction of Th1 cytokines produced by splenocytes, while promoting the sIgA secretion in the intestine [87]. *L. paracasei* KBL382 alleviates AD in mice by decreasing the production of Th1, Th2, and Th17-type cytokines in the skin tissue, increasing the production of the anti-inflammatory cytokines IL-10 and transforming growth factor-β (TGF-β), and increasing the number of regulatory T cells (Treg) in the mesenteric lymph nodes of AD mice [88]. *L. sake* WIKIM30 also played the same role in animal experiments for the treatment of AD [89]. In addition, *B. longum* CCFM1029, on the other hand, can alleviate allergic symptoms in mice by upregulating tryptophan metabolism and producing I3C to activate AHR-mediated immune responses and suppress abnormal TH2-type immune responses [90].

### 5.2. Treatment of Allergic Diseases with LAB

The effect of LAB on food protein allergies in infants is the most studied and supported by clinical data [91]. The use of LGG in infants with cow’s milk allergy before 1 year of age reduced symptoms of bloody stools, diarrhea, irritability, bloating, mucus-like stools, and vomiting, but the study also showed no effect of LGG on abdominal pain, constipation, and inflammatory skin symptoms [92]. Supplementation of LGG with eHCF was able to reduce the symptoms of blood in stool caused by cow’s milk allergy in infants [93]. The supplementation of LGG also contributes to the acquisition of oral immunity in infants, and in a dietary intervention with hydrolyzed milk powder containing LGG in infants with suspected cow’s milk allergy, it was found that the group receiving eHCF + LGG achieved 78.9% cow’s milk tolerance after 12 months of treatment, which was higher than the control group [94].

Supplementation of *B. lactis* can support the anti-allergic properties of deeply hydrolyzed formula powders, with significant relief of diarrhea and vomiting in children after addition [95]. Meanwhile, combined LAB are often used to regulate allergic diseases, and by compounding the strains, better allergy regulation can be achieved. The combination of *L. salivarius*, *B. longum*, and *L. rhamnosus* has immunomodulatory and anti-allergic effects [96]. The use of probiotics may also improve the safety and efficacy of other treatments. The results of oral immunotherapy in 201 peanut-allergic children showed that the combination of probiotic *L. rhamnosus* GG with oral immunotherapy improved the safety of oral treatment and reduced the incidence of adverse events after correction [97]. In the treatment of mice models of food allergy by combined IgE neutralization, the combination of fusion protein with *B. longum* is known to be more effective in alleviating the allergic response in food-allergic mice, further reducing mast cell numbers and free IgE levels and inhibiting mast cell proliferation [98].

A survey of 40,614 patients in Norway showed that children who consumed LAB dairy products were less likely to develop allergic eczema than controls [99]. In the treatment of AD or eczema, the common probiotic LGG is not effective in preventing eczema when used alone [100,101]. However, the addition of LGG to a combination of bacteria may have a beneficial effect in reducing eczema morbidity and improving symptoms. A follow-up study found that continuous use of LGG and *B. subspecies* BB-12 for six months was effective in reducing the incidence of eczema in infants [102]. Patients with AD in childhood and adolescence showed a significant improvement in clinical response after six months of mixed probiotic therapy (*L. rhamnosus* HN001, *L. acidophilus* NCFM, *L. paracasei* Lcp-37, and *B. lactis* HN019) [103]. When probiotic interventions were used in children, a significant reduction in the SCORAD score was observed in children receiving probiotics compared to controls. However, the treatment effect was significant in children or adolescents aged 1–18 years, but not in infants and children younger than 1 year [104]. 

The perinatal period is also a prime time for LAB supplementation. Maternal supplementation during pregnancy with LGG and *L. listeriae* LC705, *B. shortum* BB99, and *P. felis* prevents the development of allergic diseases, including eczema and asthma and other allergic diseases, in offspring born by caesarean section [105]. In a study of 474 mother–infant pairs, intervention with *L. rhamnosus* HN001 during the perinatal period of the mother and early in the life of the offspring was associated with a significant reduction in the risk of allergy in the offspring, and the incidence of eczema was significantly lower in all offspring of the *Lactobacillus* intervention up to the age of 11 years [106]. Some newly developed LAB have shown promising properties in animal trails, while *L. paracasei* KBL382 improved transcutaneous water loss, spleen/body weight ratio, scratching behavior, and IgE levels in mice with AD [88]. *L. sake* WIKIM30 reversed the gut flora of AD mice and ameliorated AD skin lesions [89].

As infant asthma and allergic rhinitis are not as common as eczema and food allergies, there are fewer studies on probiotic interventions for these two conditions. In interventions for asthma in children and adults, the combination of *L. salivarius* LS01 and *B. shortum* B632 reduced the number of asthma exacerbations and the number of children with more than two asthma exacerbations [107]. *B. infantis* 35624 in adult asthmatics increases lung capacity and reduces the frequency of rescue inhaler use [108]. LGG also has the potential to improve asthma in infants and children, and animal studies have shown that LGG can prevent the onset of allergic asthma in mice through colonization of the upper respiratory tract [77]. In the prevention and treatment of asthma in infants, a small sample size study showed that daily supplementation with LGG after birth in infants at high risk of asthma alleviated the delayed development of intestinal flora diversity, a bias in flora function towards glycolysis, and a lack of multiple anti-inflammatory lipid metabolites in high-risk infants [109]. 

Several reports have demonstrated the role of probiotics in rhinitis in mice experience and in the clinical trials among children and adults. In an intervention targeting allergic rhinitis, oral administration of *L. paracasei* GM-080 was able to reduce airway inflammation and alleviate sneezing symptoms in children with allergic rhinitis in highly airway-responsive mice [110]. Daily intake of *B. longum* and the *L. plantarum* mixture NVP-1703 for four consecutive weeks significantly reduced house-dust-mite-specific IgE levels and significantly increased serum IL-10 levels in adult patients [111]. In addition, probiotic treatment significantly reduced the use of antihistamines and topical corticosteroids in a double-blind trial of *B. lactis* BB12 and *E. faecalis* L3 given to children with allergic rhinitis [112].

Although LAB with beneficial functions have been widely used in the adjunctive treatment of allergy relief in infants and children, there are still many contradictory and ambiguous conclusions about the treatment of probiotics due to the design and operation of clinical trials [113], the conditions of the volunteers [113], and the differences in the subject strains [114], which have led to contradictory, ambiguous, and controversial conclusions [115]. Only some probiotics have shown good alleviating and preventive properties for allergic diseases, and there is still a need for further exploration of probiotics that can alleviate allergic symptoms in clinical tests [116].

## 6. Conclusions

The status of allergic diseases in infants is closely related to structural and metabolic differences in the host microbiota, with the most affected microbiota being the breast milk flora and the infant gut flora. Therefore, prospective analysis, regulation, and monitoring of either the maternal or infant flora may serve as a beneficial potential regulatory tool to prevent and alleviate allergies. Meanwhile, the use of LAB for the regulation of the microbiota and associated immune dysfunction in infants with allergic diseases is a safe and promising avenue for the treatment of infant allergic diseases. In the future, more treatments for allergic diseases in infants are expected to be discovered through modulation of the host flora.

## Data Availability

Not applicable.
